# H3K4me2 functions as a repressive epigenetic mark in plants

**DOI:** 10.1186/s13072-019-0285-6

**Published:** 2019-07-02

**Authors:** Yuhao Liu, Kunpeng Liu, Liufan Yin, Yu Yu, Ji Qi, Wen-Hui Shen, Jun Zhu, Yijing Zhang, Aiwu Dong

**Affiliations:** 1 State Key Laboratory of Genetic Engineering, Collaborative Innovation Center of Genetics and Development, International Associated Laboratory of CNRS–Fudan–HUNAU on Plant Epigenome Research, Department of Biochemistry, Institute of Plant Biology, School of Life Sciences, Fudan University, Shanghai, 200438 China; 2 State Key Laboratory of Genetic Engineering, Collaborative Innovation Center of Genetics and Development, Department of Biochemistry, Institute of Plant Biology, School of Life Sciences, Fudan University, Shanghai, 200438 China; 3 Universite de Strasbourg, CNRS, IBMP UPR 2357, 67000 Strasbourg, France; 4 Systems Biology Center, National Heart Lung and Blood Institute, National Institutes of Health, Bethesda, MD 20892 USA; 5 National Key Laboratory of Plant Molecular Genetics, CAS Center for Excellence in Molecular Plant Sciences, Institute of Plant Physiology and Ecology, Shanghai Institutes for Biological Sciences, Chinese Academy of Sciences, Shanghai, 200032 China; 6 University of the Chinese Academy of Sciences, Beijing, 100049 China

**Keywords:** H3K4me2, ChIP-seq, RNA-seq, Gene expression, Epigenetic mark, Rice

## Abstract

**Background:**

In animals, H3K4me2 and H3K4me3 are enriched at the transcription start site (TSS) and function as epigenetic marks that regulate gene transcription, but their functions in plants have not been fully characterized.

**Results:**

We used chromatin immunoprecipitation sequencing to analyze the rice genome-wide changes to H3K4me1/H3K4me2/H3K4me3 following the loss of an H3K4-specific methyltransferase, SDG701. The knockdown of *SDG701* resulted in a global decrease in H3K4me2/H3K4me3 levels throughout the rice genome. An RNA-sequencing analysis revealed that many genes related to diverse developmental processes were misregulated in the *SDG701* knockdown mutant. In rice, H3K4me3 and H3K36me3 are positively correlated with gene transcription; however, surprisingly, the H3K4me2 level was negatively associated with gene transcription levels. Furthermore, the H3K4me3 level at the TSS region decreased significantly in the genes that exhibited down-regulated expression in the *SDG701* knockdown mutant. In contrast, the genes with up-regulated expression in the mutant were associated with a considerable decrease in H3K4me2 levels over the gene body region.

**Conclusion:**

A comparison of the genome-wide distributions of H3K4me2 in eukaryotes indicated that the H3K4me2 level is not correlated with the gene transcription level in yeast, but is positively and negatively correlated with gene expression in animals and plants, respectively. Our results uncovered H3K4me2 as a novel repressive mark in plants.

**Electronic supplementary material:**

The online version of this article (10.1186/s13072-019-0285-6) contains supplementary material, which is available to authorized users.

## Background

In eukaryotes, histone lysine (K) methylation is important for regulating gene expression. The K-residues at the N-terminus of histone H3 or H4 can be mono-, di-, or tri-methylated, and the methylation of a specific K-residue as well as the number of methyl groups added can mark different chromatin states [1–3]. The methylations of H3K4 and H3K36 are generally associated with gene transcriptional activation, whereas the methylations of H3K9 and H3K27 are associated with the repression of gene expression [4–7].

As an evolutionarily conserved epigenetic mark, H3K4 methylations are broadly distributed in euchromatin and are associated with actively transcribed genes in animals and plants [2, 8–10]. Studies involving chromatin immunoprecipitation (ChIP)-chip and ChIP-sequencing (ChIP-seq) technologies have provided insights into the distributions and functions of H3K4me1/H3K4me2/H3K4me3 in various species. In yeast (*Saccharomyces cerevisiae*), H3K4me1 and H3K4me2 appear at about 600 base pairs (bp) downstream of the transcription start site (TSS), whereas H3K4me3 is primarily enriched at 200 bp downstream of the nucleosome-free region [11]. In animals, H3K4me1/H3K4me2/H3K4me3 is enriched on both sides of the TSS, with a similar distribution pattern across the genome [12–14]. Additionally, H3K4me1 is globally associated with enhancers [15] and H3K4me2/H3K4me3 usually positively regulates gene expression in animals [8–10]. Similarly, in Arabidopsis and rice (*Oryza sativa*), H3K4me3 is enriched close to the TSS region and is positively correlated with gene expression levels [4, 16–18]. However, there has been some inconsistency in the reported H3K4me2 distribution pattern, with some studies indicating that the distribution pattern is similar to that of H3K4me3 [4, 16], whereas other studies have suggested that H3K4me2 is broadly distributed across the gene body region, in contrast to H3K4me3 [17, 18]. Moreover, the relationship between H3K4me2 and gene expression levels in plants remains unclear [4, 16–18].

The methylation of H3K4 is catalyzed by SET domain-containing methyltransferases. In yeast, Set1 is the sole H3K4-specific methyltransferase [19, 20] and functions as part of the COMPASS (complex proteins associated with Set1) complex together with the other subunits, Swd1, Swd2, Swd3, Bre2, Sdc1, Spp1, and Shg1 [21, 22]. *Drosophila melanogaster* contains three Set1 homologs [dSet1, Trithorax (Trx), and Trx-related protein], all of which can associate with the other subunit proteins to form COMPASS-like complexes, which are responsible for the methylation of H3K4 [23]. Mammals have six Set1 homologs [SET1A, SET1B, MLL1, MLL2, MLL3, and MLL4] [8], which also usually exist in COMPASS-like complexes for methylating H3K4 [24–26]. In Arabidopsis, ATX1–5 function as H3K4 methyltransferases. Additionally, ATX1 is involved in root, leaf, and floral organ development [27–30]. Previous studies revealed that ATX1 and ATX2 are functionally diverse, with ATX2 exhibiting H3K4me2 rather than H3K4me3 activity [31, 32]. Moreover, ATX3–5 has redundant functions affecting vegetative and reproductive developmental processes and the genome-wide distribution of H3K4me2 and H3K4me3 [33]. In rice, SDG723/OsTrx1 exhibits histone H3 methyltransferase activity in vitro and helps to regulate flowering time [34]. The OsTrx1 protein is specifically recruited to the target gene *Ehd1* to promote rice flowering by interacting with a transcription factor, SDG723/OsTrx1/OsSET33 Interaction Protein 1 (SIP1) [35]. SDG701 is likely a major H3K4-specific methyltransferase in rice, thereby contributing to the regulation of multiple processes during rice plant growth and development, such as flowering, gametophyte development, and grain production [36].

In this study, we functionally characterized the H3K4 methylation marks (H3K4me1, H3K4me2, and H3K4me3) in rice by analyzing the *SDG701* knockdown mutant in RNA-seq and ChIP-seq assays, for the *SDG701* knockout mutant is lethal [36]. The SDG701 deficiency decreased the global H3K4me2/H3K4me3 levels *in planta* and resulted in the misregulation of many genes involved in multiple developmental processes. Interestingly, unlike the H3K4me1/H3K4me3 and H3K36me3 marks, which were positively correlated with gene transcription, H3K4me2 was negatively correlated with gene expression. Moreover, H3K4me2 levels were considerably decreased in the genes that exhibited up-regulated expression in the *SDG701* knockdown mutant, further supporting a novel function of H3K4me2 to repress gene expression in rice.

## Results

### SDG701 is required for the proper expression of many genes involved in diverse developmental processes

**Fig. 1:**
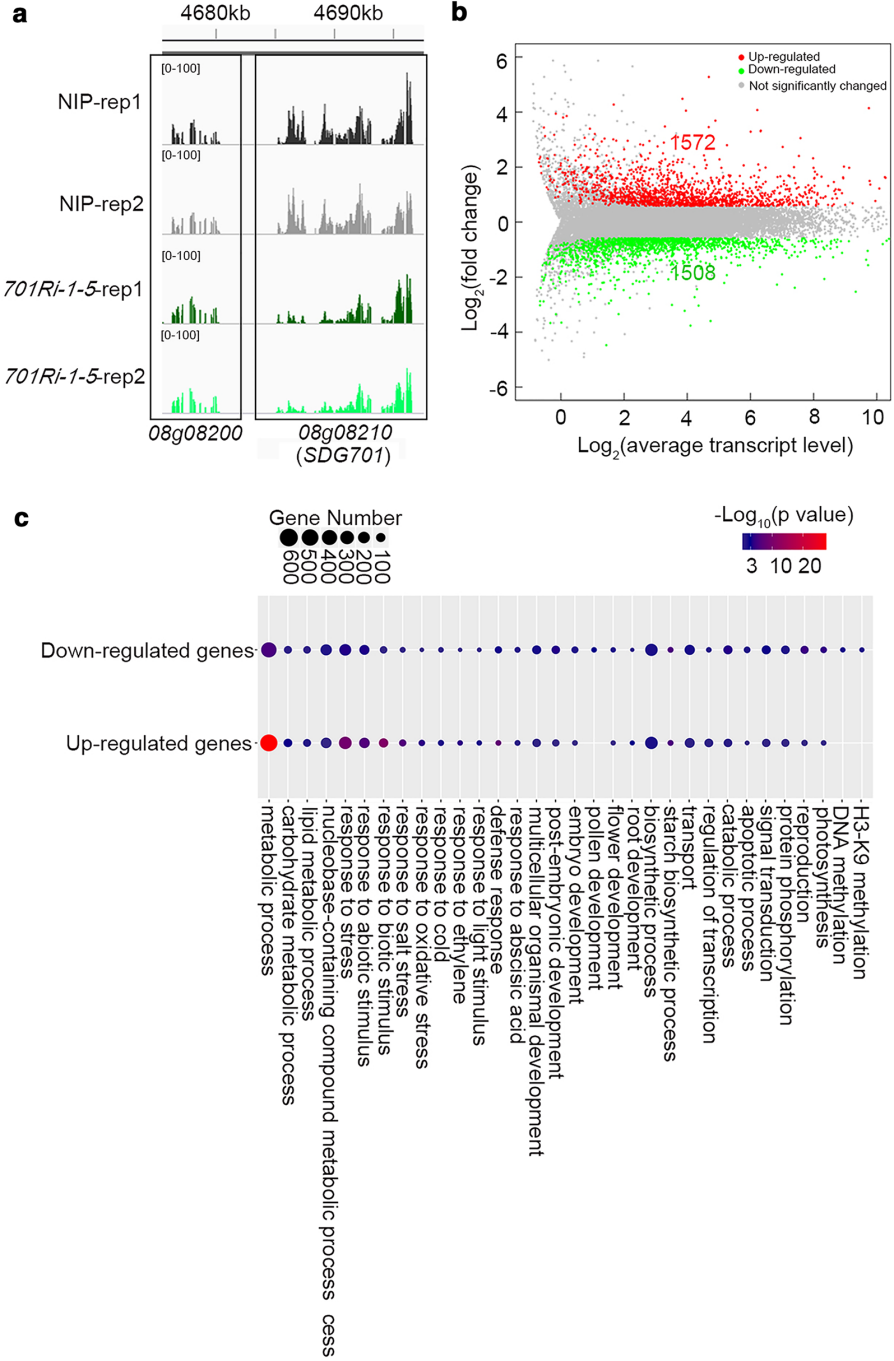
Transcriptome analysis of the *SDG701* knockdown mutant. **a** IGV screen shots presenting the normalized read densities of *LOC_Os08g08210* (*SDG701*) and *LOC_Os08g08200* (gene neighboring *SDG701*) based on an RNA-sequencing analysis of two biological replicates of the wild-type NIP and *701Ri*-*1*-*5* mutant plants. **b** MA plot presenting gene transcription changes following the knockdown of *SDG701*. Green and red points represent the genes exhibiting down- and up-regulated expression (> 1.5-fold change), respectively, whereas the gray points represent the genes with unchanged expression, in the *701Ri*-*1*-*5* mutant compared with the wild-type NIP. The adjusted *p* value (p.adj) was calculated with the Benjamini–Hochberg correction. **c** Functional enrichment analysis of genes with significantly down- or up-regulated expression levels. The size of each point represents the number of genes, and *p* values are indicated with various colors

We previously reported SDG701 functions as a major H3K4-specific methyltransferase that is important for rice plant development [36]. To further clarify the function of SDG701, we conducted an RNA-sequencing (RNA-seq) analysis of the *SDG701* knockdown mutant *35Sp::SDG701iR1* (hereafter referred to as *701Ri*-*1*-*5*) in the *O. sativa* spp. *japonica* cv. Nipponbare (NIP) background. After sequencing two biological replicates of RNA-seq libraries, more than 20 million mapped reads were obtained for a subsequent evaluation of genes that were differentially expressed between the *701Ri*-*1*-*5* mutant and the wild-type NIP (Additional file 1: Table S1). As expected, the transcription level of *SDG701* (*LOC_Os08g08210*) decreased to half in the *701Ri*-*1*-*5* mutant (FPKM_*701Ri*-*1*-*5*_/FPKM_NIP_ = 0.50, adjusted *p* value = 5.72e−4), while that of *LOC_Os08g08200*, a gene adjacent to *SDG701*, was unaffected (Fig. 1a). We previously demonstrated that SDG701 influences the expression of the flowering genes *Hd3a*, *Ehd1*, and *RFT1* [36]. Thus, the RNA-seq data were analyzed to determine the expression levels of these genes. Consistent with our previous results [36], the *Hd3a*, *Ehd1*, and *RFT1* expression levels were significantly lower in the *701Ri*-*1*-*5* mutant than in the wild-type NIP (Additional file 1: Fig. S1).

An analysis of the RNA-seq data revealed 1572 and 1508 genes whose transcription levels were up- and down-regulated by more than 1.5-fold in the *701Ri*-*1*-*5* mutant, respectively (Fig. 1b, Additional file 2: Table S2). A gene ontology (GO) enrichment analysis indicated that the misregulated genes in the *701Ri*-*1*-*5* mutant were enriched with multiple biological process GO terms, including metabolic process, biosynthetic process, response to biotic or abiotic stimuli, organ development, and signal transduction (Fig. 1c, Additional file 3: Table S3). Additionally, some of the misregulated genes in the *701Ri*-*1*-*5* mutant were related to flower development, the starch biosynthetic process, and pollen development (Additional file 3: Table S3), which is consistent with the fact that SDG701 affects gametophyte development and grain production [36]. These results imply that SDG701 is required for the proper expression of many genes and is crucial for multiple developmental processes in rice.

### H3K4me2 enrichment is negatively correlated with transcription levels in rice

**Fig. 2:**
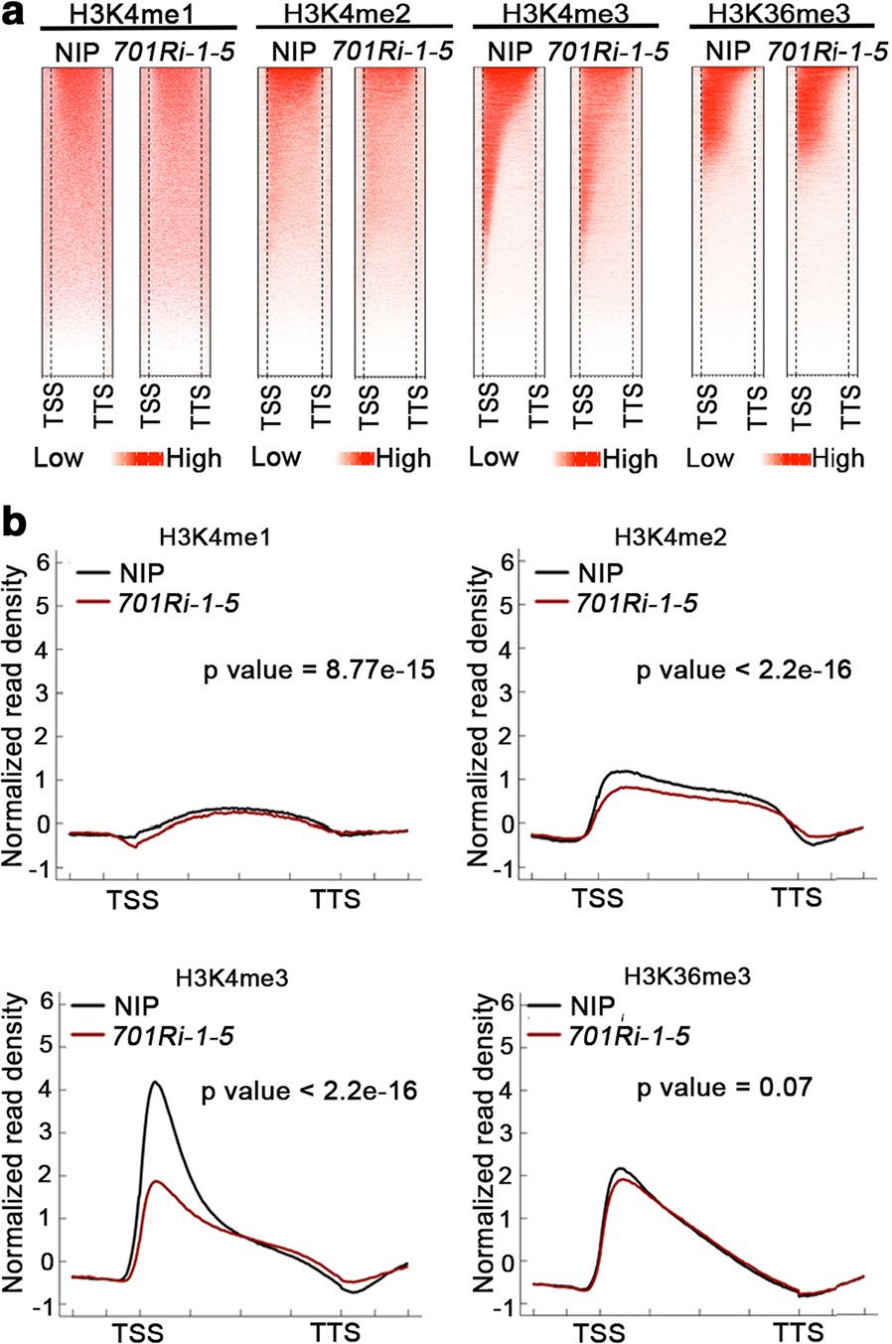
Knockdown of *SDG701* results in a global decrease in H3K4me2/H3K4me3 levels in rice. **a** Heatmaps presenting H3K4me1/H3K4me2/H3K4me3 and H3K36me3 levels surrounding genes in the *701Ri-1-5* mutant and wild-type NIP. Each line in a heatmap represents a gene. The plots were generated from 1 kb upstream of the transcription start site (TSS) to 1 kb downstream of the transcription termination site (TTS). **b** Average density plots presenting the genome-wide (on 55,986 annotated rice genes) distributions of H3K4me1/H3K4me2/H3K4me3 and H3K36me3 in the *701Ri-1-5* (red) mutant and wild-type NIP (black). The *p* values were calculated by the Kolmogorov–Smirnov test

In rice, SDG701 is an H3K4-methyltransferase that is especially important for the tri-methylation of H3K4, and the resulting H3K4me3 serves as an active epigenetic mark for gene transcription in eukaryotes. We were interested in why many genes exhibited increased expression in the *SDG701* knockdown mutant. We first performed a ChIP-seq analysis to assess whether the knockdown of *SDG701* affected H3K4 methylations. Approximately 4–20 million unique reads were obtained for H3K4me1, H3K4me2, H3K4me3, and H3K36me3 in the *701Ri*-*1*-*5* mutant and wild-type NIP (Additional file 1: Table S4 and Fig. 2a). In NIP, 284 (0.5% of 55,986 annotated rice genes), 10,681 (19.1%), and 25,259 (45.1%) genes enriched with H3K4me1, H3K4me2, and H3K4me3, respectively, were identified (Additional file 1: Fig. S2A). Another 15,728 genes enriched with H3K36me3 were also detected. The peaks for enriched H3K4me2, H3K4me3, and H3K36me3 overlapped previously published ChIP-seq data (Additional file 1: Fig. S2B–D) [37–39]. As shown in Fig. 2, H3K4me1 was distributed over the gene body region at a much lower level than H3K4me2 and H3K4me3, H3K4me2 covered the entire gene body region with a weak peak close to the TSS, whereas H3K4me3 showed a sharp peak at the TSS in the wild-type NIP. As a control, H3K36me3 was mainly distributed at the 5′ end of the gene body, which was consistent with our published data [40].

In the *SDG701* knockdown mutant, the H3K4me1 level decreased slightly, especially close to the TSS region, while the H3K4me2 and H3K4me3 levels decreased obviously (Fig. 2b). The ChIP-seq data further confirmed that SDG701 is responsible for methylating H3K4, especially the di- and tri-methylation of H3K4 in rice (Fig. 2). We observed that the H3K36me3 level decreased very slightly close to the TSS in the *701Ri*-*1*-*5* mutant (Fig. 2b). We wondered whether this decrease was directly due to the knockdown of *SDG701*. Because the H3K4me1-enriched genes accounted for 0.5% of all annotated rice genes, we only selected H3K4me2-, H3K4me3-, and H3K36me3-enriched genes for further analyses. These genes were grouped into the following seven clusters based on their surrounding epigenetic marks (Additional file 1: Fig. S3A): (a) enriched with only H3K4me2, (b) enriched with only H3K4me3, (c) enriched with only H3K36me3, (d) enriched with H3K4me2 and H3K4me3, (e) enriched with H3K4me2 and H3K36me3, (f) enriched with H3K4me3 and H3K36me3, and (g) enriched with H3K4me2, H3K4me3, and H3K36me3. Heatmaps and average density plots were generated for these genes in NIP and the *701Ri*-*1*-*5* mutant (Additional file 1: Fig. S3B–E). The H3K4me2 and H3K4me3 signals were significantly decreased for the genes in clusters a and b, respectively; however, the enrichment of H3K36me3 in the genes of cluster c was unaffected, indicating that SDG701 did not directly contribute to the decrease in the H3K36me3 level in the *701Ri*-*1*-*5* mutant. Regarding the genes in cluster f (enriched with H3K4me3/H3K36me3) and cluster g (enriched with H3K4me2/H3K4me3/H3K36me3), the H3K36me3 signal decreased slightly close to the TSS, implying that the decrease in the H3K36me3 level in the *701Ri*-*1*-*5* mutant was probably related to a decrease in the H3K4me3 level. These results suggested that the knockdown of *SDG701* in rice leads to a genome-wide decrease in H3K4 methylations, especially the di- and tri-methylations.

**Fig. 3:**
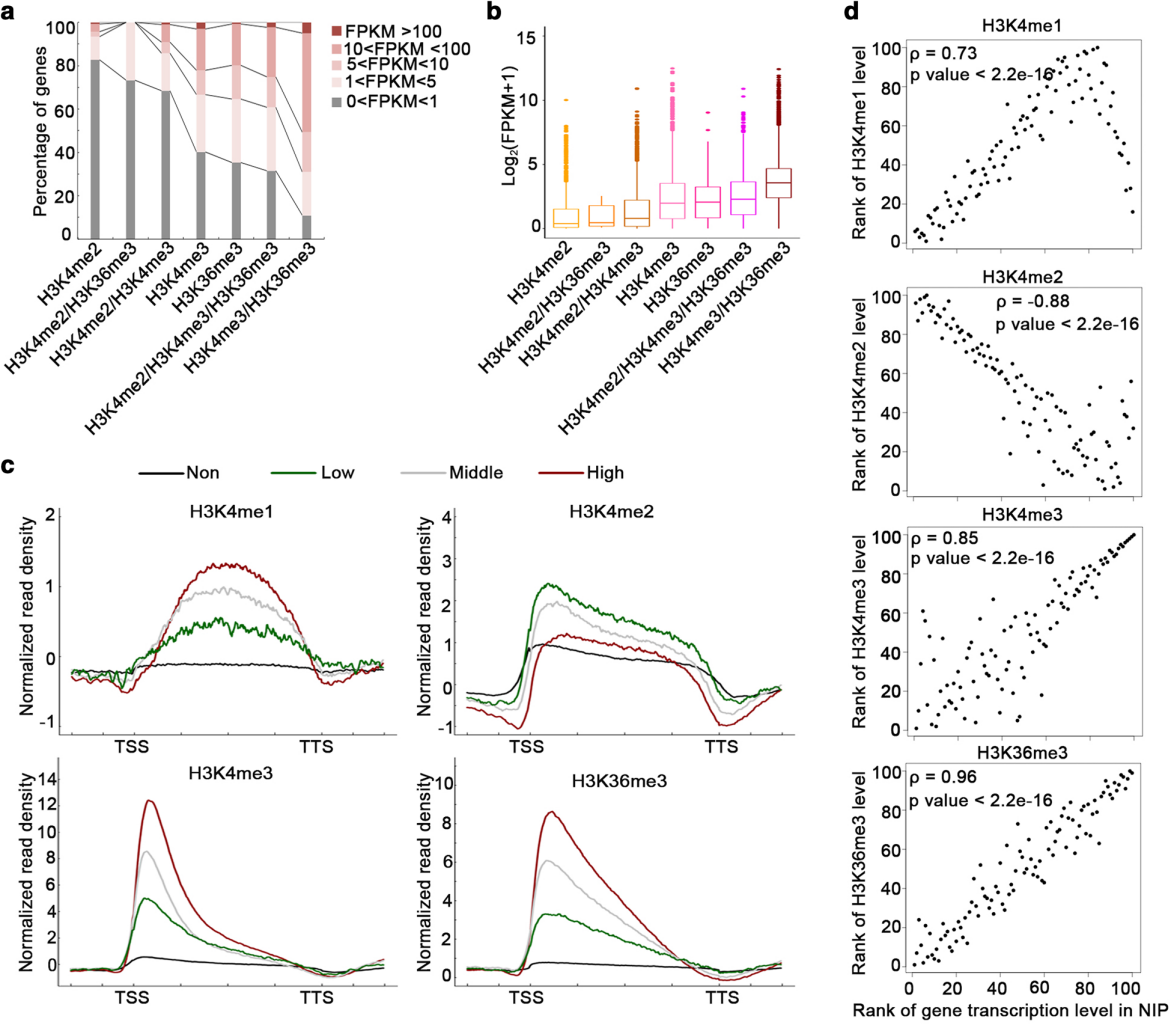
H3K4me2 level is negatively correlated with the gene transcription level in rice. **a** Genes marked by H3K4me2 only, H3K4me3 only, H3K36me3 only, H3K4me2 and H3K4me3, H3K4me2 and H3K36me3, or H3K4me2, H3K4me3, and H3K36me3 exhibited varying transcription levels in the wild-type NIP. Genes categorized by transcription levels (FPKM values) are distinguished by different colors. **b** Box plots presenting the transcription levels of the genes with a differentially marked H3 in (**a**) (*p* < 0.001 according to the Wilcoxon test). **c** Average density plots presenting the H3K4me1/H3K4me2/H3K4me3 and H3K36me3 distribution patterns along the differentially expressed genes in the wild-type NIP. The plots were generated from 1 kb upstream of the TSS to 1 kb downstream of the TTS. The annotated rice genes were grouped based on transcription levels. Non: not expressed, 0 < FPKM < 1 (black); Low: low expression level, 1 < FPKM < 2 (green); Middle: moderate expression level, 2 < FPKM < 10 (gray); and High: high expression level, FPKM > 10 (red). **d** Scatter plots presenting the relationship between H3K4me1/H3K4me2/H3K4me3 and H3K36me3 levels and gene transcription levels in the wild-type NIP. The H3K4me1/H3K4me2/H3K4me3 and H3K36me3 (from the TSS to the TTS) levels were calculated as follows: ChIP-seq normalized read density−input normalized read density for expressed genes (FPKM > 1). Spearman’s rank correlation coefficient indicates the correlation between the methylation level and the gene expression level. The *p* value was determined based on Spearman’s rank correlation test

We subsequently explored the correlation between H3K4 methylations and transcript levels by analyzing the RNA-seq and ChIP-seq data for the wild-type NIP. About 90% of the H3K4me3/H3K36me3-enriched genes (cluster f) were expressed (FPKM > 1; Fig. 3a). Unexpectedly, over 80% of the H3K4me2-enriched genes (cluster a) were not expressed (0 < FPKM < 1; Fig. 3a). We also observed that if gene groups were marked with H3K4me2, the fraction of expressed genes decreased. For example, approximately 60% of the H3K4me3-enriched genes were expressed, whereas only about 30% of the H3K4me2/H3K4me3-enriched genes were expressed (Fig. 3a). Similarly, more than 60% of the H3K36me3-enriched genes were expressed, while less than 30% of the H3K4me2/H3K36me3-enriched genes were expressed (Fig. 3a). Next, we analyzed the expression levels of the seven gene clusters (Fig. 3b). The mean expression level of the H3K4me3/H3K36me3-enriched genes was the highest, and that of the H3K4me2-enriched genes was the lowest (Fig. 3b, Additional file 1: Table S5). Moreover, a comparison of the seven clusters revealed that the average expression levels were obviously lower for the genes marked with H3K4me2 than for the genes that lacked H3K4me2 (Fig. 3b, Additional file 1: Table S5), suggesting that H3K4me2 is probably related to gene repression.

To further validate our results, we classified all annotated rice genes into the following four groups based on the FPKM values: Non: not expressed, 0 < FPKM < 1; Low: low expression level, 1 < FPKM < 2; Middle: moderate expression level, 2 < FPKM < 10; High: high expression level, FPKM > 10. Additionally, average density plots for H3K4me1/H3K4me2/H3K4me3 and H3K36me3 were generated for these four gene groups (Fig. 3c). A positive correlation was observed between the transcript levels and the H3K4me1, H3K4me3, and H3K36me3 levels (Fig. 3c, d). In contrast, the H3K4me2 level was negatively correlated with the transcript level, just similar as the relationship between H3K27me3 and gene transcription (Additional file 1: Fig. S4). Specifically, H3K4me3 (*ρ* = 0.85) and H3K36me3 (*ρ* = 0.96) levels were positively correlated with gene transcription levels. A weaker positive correlation was detected between H3K4me1 levels (*ρ* = 0.73) and gene transcription levels. Consistent with the results for H3K4me2 in NIP (*ρ* = -0.88), we detected a negative correlation between gene transcription levels and H3K4me2 enrichment in another rice subspecies, *O. sativa* ssp. *japonica* cv. Dongjin (*ρ* = − 0.89; Additional file 1: Fig. S5). Our results proved that unlike H3K4me3 and H3K36me3 modifications, H3K4me2 is negatively correlated with transcript level in rice.

### H3K4me2 enrichment significantly decreases within the chromatin of the up-regulated genes in *SDG701* knockdown mutant

**Fig. 4:**
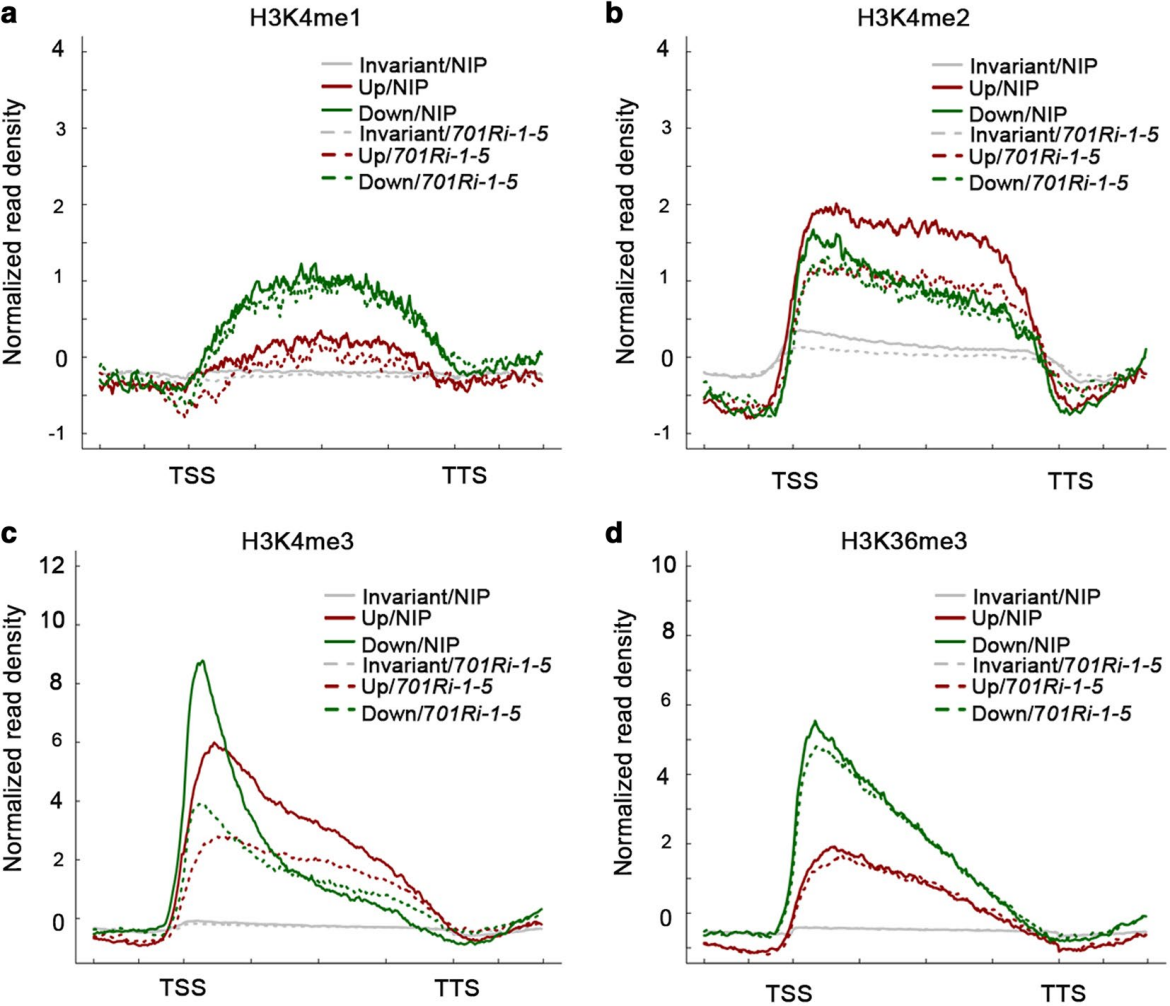
Genes exhibiting up-regulated expression in the *701Ri*-*1*-*5* mutant have decreased H3K4me2 levels. **a**–**d** Average density plots presenting the H3K4me1/H3K4me2/H3K4me3 and H3K36me3 distribution patterns along genes exhibiting significantly up-regulated expression (up: 1572 genes with expression levels > 1.5-fold higher in the *701Ri*-*1*-*5* mutant than in the wild-type NIP), down-regulated expression (down: 1508 genes with expression levels > 1.5-fold lower in the *701Ri*-*1*-*5* mutant than in the wild-type NIP), and no significant changes (Invariant) in the NIP (solid line) and *701Ri*-*1*-*5* mutant (dashed line) samples. The plots were generated from 1 kb upstream of the TSS to 1 kb downstream of the TTS

To further test the correlation between H3K4 methylations and gene transcription, we analyzed the H3K4me1/H3K4me2/H3K4me3 and H3K36me3 signals for the misregulated genes in the *701Ri*-*1*-*5* mutant. Additionally, average density plots were generated to visualize the H3K4me1/H3K4me2/H3K4me3 and H3K36me3 distribution patterns on genes that exhibited significantly up-regulated (1572 genes), down-regulated (1508 genes), or unchanged expression levels in NIP and the *701Ri*-*1*-*5* mutant (Figs. 1b and 4). There were no obvious differences between NIP and the *701Ri*-*1*-*5* mutant regarding the H3K4me1 and H3K36me3 signals on the up- and down-regulated genes (Fig. 4a, d), indicating that the gene transcription changes in the *701Ri*-*1*-*5* mutant were not due to changes in H3K4me1 or H3K36me3 enrichment. The H3K4me2 level over the whole gene body regions of the 1572 up-regulated genes was significantly lower in the *701Ri*-*1*-*5* mutant than in NIP, while the corresponding level of the down-regulated genes was only slightly lower close to the TSS in the *701Ri*-*1*-*5* mutant than in NIP (Fig. 4b). We randomly selected eight genes, whose H3K4me2 levels significantly decreased and transcript levels increased, respectively, in *701Ri*-*1*-*5* compared to in NIP for independent validation. The RT-qPCR and ChIP-qPCR results further supported that the decreased H3K4me2 correlates with gene activation (Additional file 1: Fig. S6). Regarding the H3K4me3 level, a significant decrease was observed for both up- and down-regulated genes (Fig. 4c). Moreover, the H3K4me3 signal for the down-regulated genes was mainly a sharp peak close to the TSS, while that for the up-regulated genes was a smaller peak around the TSS and extended to the entire gene body region. Our data indicated that the genes exhibiting up-regulated expression in the *701Ri*-*1*-*5* mutant had a significantly decreased H3K4me2 level, providing further evidence that H3K4me2 represses gene transcription in rice.

### Correlation between H3K4me2/H3K4me3 deposition and gene transcription

**Fig. 5:**
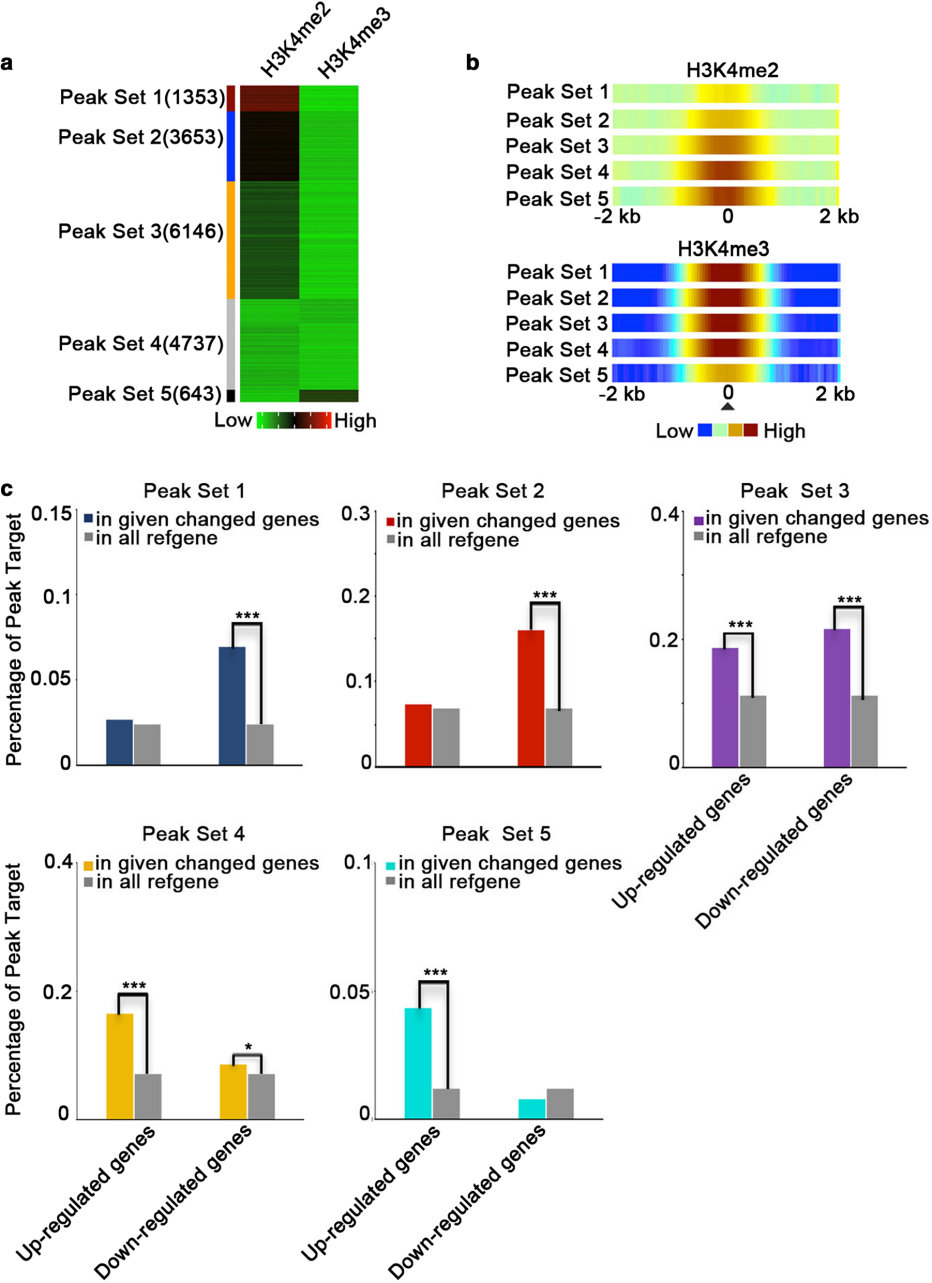
Decreases in H3K4me2 and H3K4me3 levels result in up- and down-regulated gene expression, respectively. **a** Heatmap for five sets of peaks based on the difference in the H3K4me2/H3K4me3 levels between the *701Ri*-*1*-*5* mutant and the wild-type NIP. Significant changes to H3K4me2 and H3K4me3 peaks were based on an FDR value < 1e−4 and > 1.5-fold change (*701Ri*-*1*-*5*/NIP ≤ 1.5). Green, black, and red represent the peaks with decreased, unchanged, and increased H3K4me2 and H3K4me3 levels in the *701Ri*-*1*-*5* mutant relative to the levels in the wild-type NIP, respectively. **b** Visualization of the average normalized read intensity profile close to the H3K4me2/H3K4me3 peak center (marked as 0) for each peak set in wild-type NIP. **c** Enrichment analysis of the target genes of Peak Set 1–5 among the genes exhibiting up- or down-regulated expression in the *701Ri*-*1*-*5* mutant. ***p < 1e−4 and *p < 0.05 according to Fishers’ exact test

To clarify the relationship between H3K4me2/H3K4me3 enrichment and gene transcription levels regulated by SDG701, we screened for genes with H3K4me2 or H3K4me3 levels that were significantly lower in the *701Ri*-*1*-*5* mutant (> 1.5-fold) than in NIP. We detected 1885 peaks and 14,647 peaks, corresponding to 1968 of 10,681 H3K4me2-enriched (19%) and 14,473 of 25,259 H3K4me3-enriched (57.3%) genes, respectively, with significantly lower H3K4me2 or H3K4me3 levels in the *701Ri*-*1*-*5* mutant than in NIP (Additional file 1: Fig. S7 and Additional file 4: Table S6). We subsequently calculated the read density change for each peak and clustered the peaks into the following five sets with a K-means clustering algorithm (Fig. 5a): Peak Set 1 containing 1353 peaks with a slightly increased H3K4me2 level and a significantly decreased H3K4me3 level, Peak Set 2 containing 3653 peaks with an unchanged H3K4me2 level and a significantly decreased H3K4me3 level, Peak Set 3 containing 6146 peaks with a slightly decreased H3K4me2 level and a significantly decreased H3K4me3 level, Peak Set 4 containing 4737 peaks with significantly decreased H3K4me2 and H3K4me3 levels, and Peak Set 5 containing 643 peaks with a significantly decreased H3K4me2 level and an unchanged H3K4me3 level. In the wild-type NIP, the H3K4me2 levels were higher for the genes in Peak Sets 4 and 5 than for the genes in Peak Sets 1–3, and the H3K4me3 levels were higher for the genes in Peak Sets 1–4 than for the genes in Peak Set 5 (Fig. 5b). Next, we conducted an enrichment analysis of the genes in Peak Sets 1–5 and the genes that exhibited up- or down-regulated expression in the *701Ri*-*1*-*5* mutant. The corresponding genes of Peak Set 1 and Peak Set 2 were more significantly enriched in the genes with down-regulated expression than those with up-regulated expression in *701Ri*-*1*-*5* mutant (Fig. 5c). In these two peak sets, only H3K4me3 levels were significantly decreased in the *701Ri*-*1*-*5* mutant (Fig. 5a), suggesting that the decrease in the H3K4me3 level is primarily responsible for down-regulating gene expression in rice. Meanwhile, the genes of Peak Set 5 were more significantly enriched in the genes with up-regulated expression in *701Ri*-*1*-*5* mutant (Fig. 5c), indicating that the decrease in the H3K4me2 level is mainly related to gene activation in rice. Thus, H3K4me3 and H3K4me2 are apparently positively and negatively associated with rice gene transcription, respectively. Regarding the genes of Peak Sets 3 and 4, we observed that for the genes with up-regulated expression, H3K4me2 and H3K4me3 were mainly distributed over the whole gene body, whereas for the genes with down-regulated expression, H3K4me2 and H3K4me3 produced a peak close to the TSS in NIP, especially the genes of Peak Set 4 (Additional file 1: Fig. S8). These results suggested that the relative positions of H3K4me2 and H3K4me3 along genes probably also contribute to the regulation of gene expression.

### Evolutionary diversity of H3K4me2 distributions and functions in various species

**Fig. 6:**
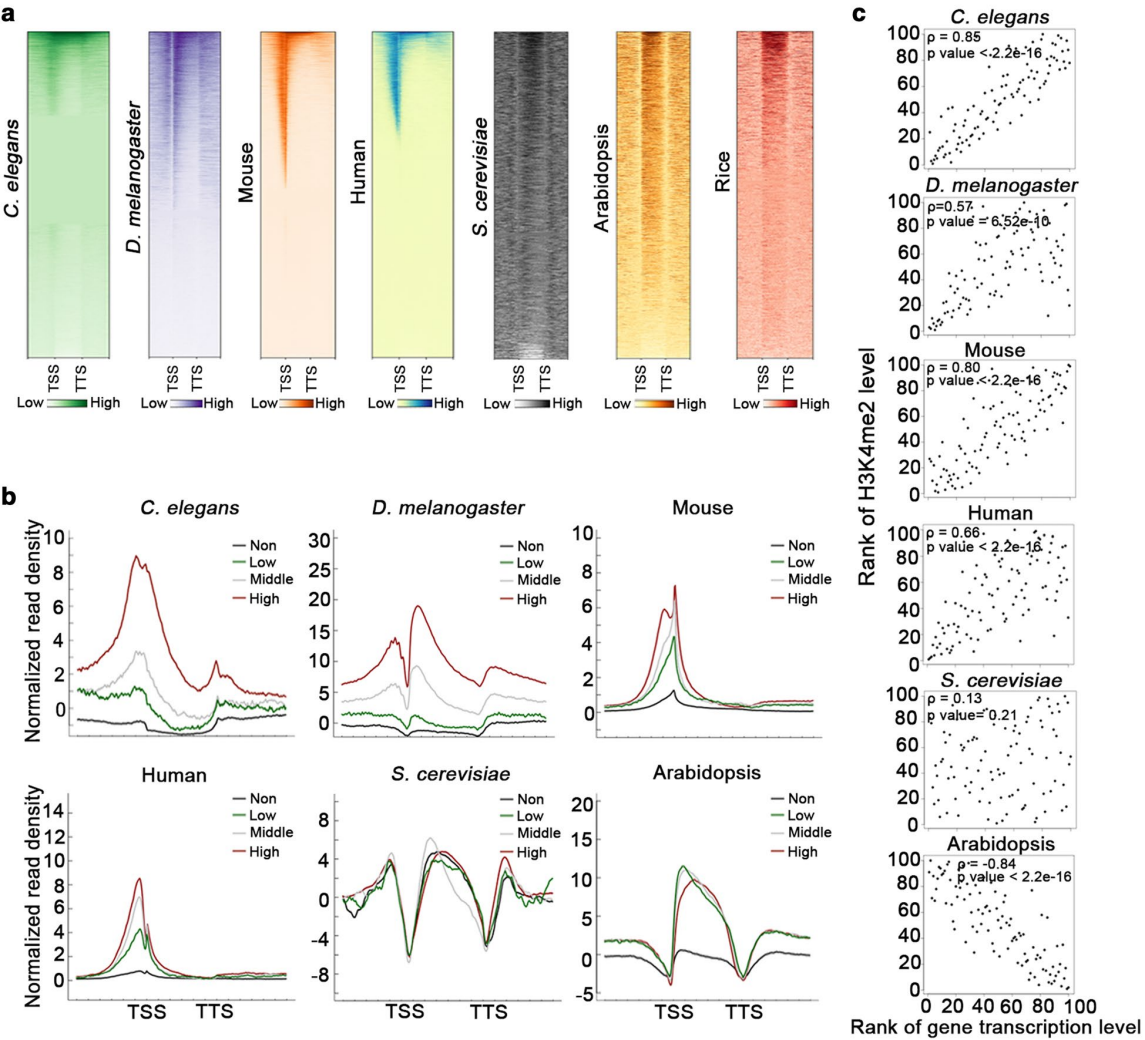
H3K4me2 distribution patterns and functions vary in yeast, animals, and plants. **a** Heatmaps presenting the genome-wide H3K4me2 distributions in various species. The plots were generated from 3 kb upstream of the TSS to 3 kb downstream of the TTS. From left to right: *C. elegans*, *D. melanogaster*, mouse embryonic stem cells (mESCs), human colon cancer cell line (HCT116 cells), *S. cerevisiae*, Arabidopsis, and rice. **b** Average density plots presenting the H3K4me2 distributions along differentially expressed genes in various species. The plots were generated from 3 kb upstream of the TSS to 3 kb downstream of the TTS. (Non: 0 < FPKM < 1; Low: 1 < FPKM < 2; Middle: 2 < FPKM < 10; and High: FPKM > 10). **c** Scatter plots presenting the relationship between the H3K4me2 level and the gene transcription level. The H3K4me2 levels were calculated as follows: ChIP-seq normalized read density—input normalized read density for expressed genes (FPKM > 1). Spearman’s rank correlation coefficient indicates the correlation between the methylation level and the gene expression level. The *p* value was determined based on Spearman’s rank correlation test

The negative correlation between H3K4me2 enrichment and gene transcription levels compelled us to analyze the H3K4me2 distribution in various species. Heatmaps (Fig. 6a) and average density plots (Fig. 6b) of H3K4me2 were generated for genes from 3 kb upstream of the TSS to 3 kb downstream of the transcription termination site (TTS). We determined that H3K4me2 was mainly enriched from 500 bp upstream of the TSS to 500 bp downstream of the TTS in *S. cerevisiae*, but was not obviously correlated with gene transcription (Fig. 6a–c), which is consistent with published data [41]. In *Caenorhabditis elegans*, H3K4me2 was primarily enriched around the TSS and was positively correlated with gene expression (Fig. 6a–c). In *D. melanogaster*, the H3K4me2 levels on each side of the TSS were also positively correlated with gene expression (Fig. 6a–c). In a human colon cancer cell line (HCT116 cells) and mouse embryonic stem cells, H3K4me2 was sharply enriched around the TSS and was positively correlated with gene expression (Fig. 6a–c). Similar to rice, the H3K4me2 in Arabidopsis was mainly enriched in gene body regions and was negatively related to gene expression (Fig. 6a–c). Unlike H3K4me2, H3K4me3 exhibited a conserved positive correlation with gene transcription in various species (Additional file 1: Fig. S9). Thus, the H3K4me2 distribution patterns are diverse among various species. Moreover, in contrast to in animals, H3K4me2 exhibits a unique distribution pattern and functions as a repressive epigenetic mark in plants.

## Discussion

In this study, we observed that the genome-wide levels of H3K4 modifications, especially H3K4me2 and H3K4me3, were significantly lower in the *SDG701* knockdown mutant than in the wild-type NIP. Our RNA-seq analysis revealed that the knockdown of *SDG701* results in more genes with up-regulated expression than genes with down-regulated expression, which is inconsistent with the fact that SDG701 functions as an H3K4-specific methyltransferase that catalyzes the epigenetic modifications that activate gene expression. Surprisingly, our combined analysis of ChIP-seq and RNA-seq data indicated that in rice, H3K4me2 enrichment is negatively correlated with gene transcript level, which differs from the positive association between H3K4me3 and H3K36me3 levels and gene expression levels. In the *SDG701* knockdown mutant, the down-regulated genes were associated with a decreased H3K4me3 level close to the TSS region, while the up-regulated genes were associated with a decreased H3K4me2 level over the gene body region. A further analysis of published H3K4me2/me3 ChIP-seq and RNA-seq raw data for various species indicated that unlike in animals, H3K4me2 in rice and Arabidopsis specifically distributes over the gene body region and functions as a repressive epigenetic mark.

Although H3K4me2 serves as a repressive mark on chromatin in plants, it was unclear whether it is more common in euchromatin or heterochromatin. We observed that more than 90% of the genes marked with H3K4me2 are not transposable element (non-TE) genes (Additional file 1: Fig. S10A), suggesting that H3K4me2 occurs mainly on euchromatin in plants. We also analyzed the relationship between H3K4me2 and other repressive epigenetic marks in rice, including DNA methylations (CG, CHG, and CHH), H3K9me1/H3K9me2/H3K9me3, and H3K27me3 [42, 43]. We observed that the genes marked with H3K4me2 overlapped less with those marked with DNA methylations or with H3K9me1/H3K9me2/H3K9me3, but more with the genes marked with H3K27me3 (Additional file 1: Fig. S10B–F). Thus, H3K4me2 probably functions together with H3K27me3 to inhibit the expression of euchromatin genes in plants. Regarding the mechanism underlying the gene repression function of H3K4me2, one possibility is that H3K4me2 helps to recruit a repressive reader protein. Consistent with this possibility, the rice DNA-binding domain (CHD) protein CHR729 reportedly can bind to both H3K4me2 and H3K27me3 in vitro through its PHD finger domain [44]. Homologs of CHR729 (CHD3 subfamily) in *D. melanogaster* and mammals are the key subunits of NuRD complexes, which exhibit nucleosome remodeling and deacetylase activities and repress transcription [45, 46]. Future studies will need to reveal the molecular mechanisms regulating H3K4me2 functions that repress gene expression in plants.

In the *SDG701* knockdown mutant, 597 genes exhibited up-regulated expression probably due to the decrease in the H3K4me2 level. More than 30% of the 597 genes are involved in plant responses to various factors, including stress, abiotic and biotic stimuli, endogenous stimuli, cold conditions, and wounding (Additional file 5: Table S7). Additionally, SDG701-mediated H3K4me2 appears to have a substantial role in plant adaptive responses to changing environmental conditions. Specifically, SDG701 can establish two epigenetic marks with distinct functions, H3K4me3 and H3K4me2, which activate and repress transcription, respectively. The data presented herein suggest there exists a new layer of complexity to histone methylation functions regarding the remodeling of chromatin. The enhanced flexibility of the chromatin remodeling function to regulate gene expression may be advantageous for plants because of the high plasticity of their growth and development and the fact they must be able to adapt to adverse environmental conditions because they are sessile organisms.

## Conclusion

We characterized the novel function of H3K4me2 in rice: different from the positive correlation between H3K4me3 level and gene expression level, H3K4me2 level is negatively correlated with gene transcription level. A single enzyme SDG701 can establish two epigenetic marks with distinct functions, suggesting the complexity of histone methylations to enhance the flexibility of the chromatin and the plasticity of the environmental adaption in plants.

## Materials and methods

### Plant materials and growth conditions

The *SDG701* knockdown mutant *35Sp::SDG701iR1* (*701Ri*-*1*-*5*) in the *O. sativa* spp. *japonica* cv. Nipponbare background used in this study has been previously described [36]. For the RNA-seq and ChIP-seq analyses, rice seedlings were grown in growth chambers under short-day photoperiod conditions [10-h light (30 °C):14-h dark (28 °C)].

### Library construction and sequencing

To be able to compare the RNA-seq and ChIP-seq data, the same set of rice samples were used to extract the total RNA used to prepare the RNA-seq and ChIP-seq libraries. Total RNA was extracted from 30-day-old shoots with the TRIzol reagent (Invitrogen). A strand-specific RNA-seq library was constructed as previously described [47]. Additionally, ChIP assays were completed as previously described [48], with the following antibodies: anti-monomethyl-H3K4 (Millipore), anti-dimethyl-H3K4 (Millipore), anti-trimethyl-H3K4 (Millipore), and anti-trimethyl-H3K36 (Abcam, Shanghai, China). The DNA resulting from a ChIP experiment and the input control were used to construct a ChIP-seq library as previously described [6]. The sequencing of the ChIP-seq and RNA-seq libraries generated 51-bp single-end reads and 101-bp paired-end reads, respectively. Details regarding the ChIP-seq and RNA-seq mapping are summarized in Additional file 1: Tables S1 and S4, respectively.

### RNA-seq analysis

More than 30-million high-quality raw reads were obtained from the sequencing of the RNA-seq library and were analyzed as previously described [49]. Briefly, the TopHat2 (version 2.0.13) [50] and Cufflinks (version 2.2.1) [51] programs were used to identify genes that were differentially expressed between the *701Ri*-*1*-*5* mutant and NIP. Significantly differentially expressed genes were defined based on the combined thresholds of > 1.5-fold change in expression level and an adjusted *p* < 0.05. A volcano plot was subsequently generated with the ggplot2 package [52] of the R software. To visualize the RNA-seq data, the mapping files from TopHat (version 2.0.13) were converted to BEDGRAPH format files with BEDTools (version 2.17.0) [53]. The BEDGRAPH format files were analyzed with the IGV (version 2.3.88) genome browser [54].

### ChIP-seq analysis

The raw data were mapped to the reference Nipponbare (*japonica*) rice genome (MSU7; http://rice.plantbiology.msu.edu) with the Bowtie (version 0.7.15) program [55], and SAMtools was used to extract the unique and non-redundant mapped reads [56]. The non-redundant mapped reads were subsequently analyzed.

To examine the global distributions of H3K4me1/H3K4me2/H3K4me3 and H3K36me3, each gene (from the TSS to the TTS) longer than 500 bp was divided into 300 bins, and 1000 bp regions upstream of the TSS and downstream of the TTS for each gene were divided into 50 bins. The read number for each bin was calculated with BEDTools (version 2.17.0) [53], and the read coverage of each sample was normalized to 10 million reads and calibrated against the input control. All aggregated plots and heatmaps were generated with the R software. BED format files were produced from mapped files with BEDTools (version 2.17.0) [53]. Additionally, SICER.sh from the SICER (version 1.1) software [57] was used to identify histone methylation enrichment regions (peaks) by comparing the ChIP library with the input library (parameters: *W* = 200, *G* = 200, and FDR < 1e−2 for H3K4me1 and H3K4me2; *W* = 200, *G* = 600, and FDR < 1e−2 for H3K4me3 and H3K36me3). Peaks were considered significant if they satisfied the following thresholds: FDR < 1e−3 and IP DNA/input DNA ≥ 2. Moreover, SICER-df.sh was used to compare the differences in H3K4me2/H3K4me3 between the *701iR1*-*5* mutant and NIP. The following thresholds were used to determine whether peak changes were significant: FDR < 1e−4 and > 1.5-fold change (wild type/mutant ≥ 1.5). The ChIPpeakAnno package [58] from Bioconductor (http://www.bioconductor.org) was used for annotating peaks. Genes (including the 1000 bp region upstream of the TSS) that overlapped with the peaks of various histone modifications were considered as modification-enriched genes.

### Gene ontology and plant ontology analysis

For the GO enrichment analysis, we analyzed the genes that exhibited down- or up-regulated expression in the *701iR1*-*5* mutant with the CARMO online tool, which is a comprehensive annotation platform for the functional exploration of rice multi-omics data (http://bioinfo.sibs.ac.cn/carmo/Gene_Annotation.php) [59]. The significantly enriched biological processes among the differentially expressed genes (*p* < 0.05) were analyzed.

### Analysis of published data

We downloaded DNA methylation, H3K9me1/H3K9me2/H3K9me3, H3K27me3, H3K4me2/H3K4me3 ChIP-seq, and related gene transcription data for various species from the Sequence Read Archive (SRA) database (https://trace.ncbi.nlm.nih.gov/Traces/sra/sra/). The SRA files were converted to FASTQ files with the fastq-dump package in the SRAToolkit (version 2.9.0) (http://www.ncbi.nlm.nih.gov/Traces/sra/?view=toolkit_doc). Distribution plots and heatmaps were generated with deepTools (version 2.0) [60]. The gene expression matrices of the other species, except for *S. cerevisiae*, were downloaded from the GEO database. The FPKM value of each *S. cerevisiae* gene was calculated with the DESeq2 package [61]. Additionally, raw DNA methylation data were re-analyzed with BatMeth package [62]. The accession numbers of the sequences analyzed in this study are as follows: GSE95356 (H3K4me2/me3) and GSE73406 (RNA) for *S. cerevisiae* [11]; GSE49733 (H3K4me2), GSE49739 (H3K4me3), and GSE100723 (RNA) for *C. elegans* (http://www.modencode.org); GSE98967 (H3K4me2/me3) and GSE98966 (RNA) for *D. melanogaster* testis cells (http://www.modencode.org); GSE80049 (H3K4me2/3) [10] and GSE98063 (RNA) [63] for *Mus musculus* embryonic stem cells; GSE51176 (H3K4me2/3) [12] and GSE54167 (RNA) [64] for the human colon cancer cell line (HCT116 cells); GSE73972 (H3K4me2/3 and RNA) for Arabidopsis [33]; and GSE81436 (H3K9me2) [43], GSE79033 (H3K9me1//me3 and H3K27me3) [42] and GSE19602 (DNA methylations) for rice [37].

### RNA extraction and RT-qPCR analysis

According to the manufacturer’s instructions (Invitrogen), RNA was isolated with TRI Reagent using the same method described in RNA-seq. Reverse transcription was performed with Improm-II reverse transcriptase (Promega), and quantitative polymerase chain reaction (qPCR) was performed using specific primers listed in Additional file 1: Table S8, and *Ubiquitin5 (Ubq5)* was used as an internal reference gene for normalization [36].

### ChIP-qPCR analysis

Chromatin immunoprecipitation (ChIP) assays were performed with the same method described in the library construction. qPCR was used to test the enrichment of DNA immunoprecipitated in the ChIP experiments. Gene-specific primers are listed in Additional file 1: Table S8.

## Additional files

**Additional file 1.** Figures S1–S10 and Tables S1, S4, S5 and S8.

**Additional file 2: Table S2.** Genes up- and down-regulated by more than 1.5-fold in *701Ri-1-5* mutant compared to in the wild-type NIP.

**Additional file 3: Table S3.** Biological functions enriched in the up- and down-regulated genes in *701Ri-1-5* mutant compared to in the wild-type NIP.

**Additional file 4: Table S6.** Peaks and genes with decreased levels of H3K4me2 or H3K4me3 to more than 1.5-fold in *701Ri-1-5* mutant compared to in the wild-type NIP.

**Additional file 5: Table S7.** Biological functions enriched in 597 up-regulated genes with decreased H3K4me2 levels in *701Ri-1-5*.

## Data Availability

The ChIP-seq and RNA-seq data generated in this study have been deposited in NCBI BioProject database (https://www.ncbi.nlm.nih.gov/bioproject/) and are accessible through accession number PRJNA522350.

## Abbreviations

TSS: transcription start site
TTS: transcription termination site
ChIP-seq: chromatin immunoprecipitation sequencing
RNA-seq: RNA sequencing
MLL: mixed lineage leukemia
COMPASS: complex proteins associated with Set1
*Ehd1*: *EARLY HEADING DATE 1*
*Hd3a*: *HEADING DATE 3a*
*RFT1*: *RICE FLOWERING LOCUS T 1*
SIP1: SDG723/OsTrx1/OsSET33 Interaction Protein 1
NIP: *O. sativa* spp. *japonica* cv. Nipponbare
DJ: *O. sativa* ssp. *japonica* cv. Dongjin
FPKM: fragments per kilobase of exon per million reads mapped
