# Supplementary material for: H3K4me2 functions as a repressive epigenetic mark in plants
Source: Epigenetics Chromatin. 2019 Jul 2;12:40. doi: 10.1186/s13072-019-0285-6 (PMC6604379; doi:10.1186/s13072-019-0285-6)
Supplement: Supplementary file 1 — Additional file 1. Figures S1–S10 and Tables S1, S4, S5 and S8. [file 13072_2019_285_MOESM1_ESM.docx]

**
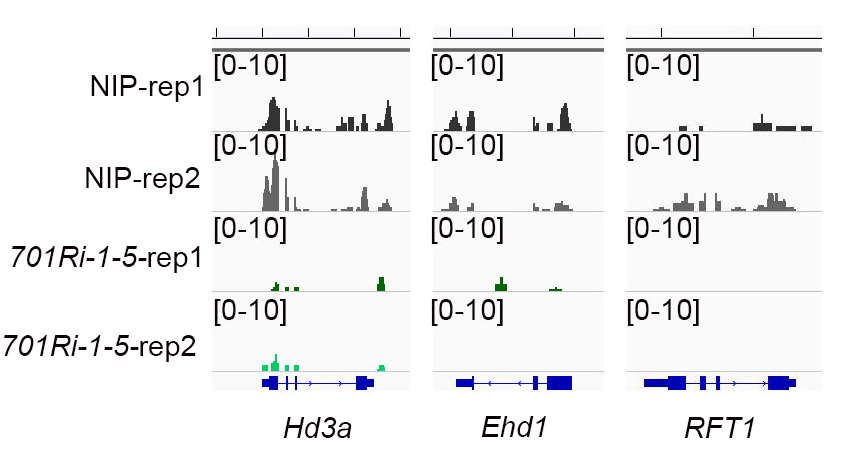
**

**Figure S1**. IGV plots indicating that *Hd3a*, *Ehd1*, and *RFT1* transcription levels were significantly lower in the *701Ri-1-5* mutant than in the wild-type NIP.

**
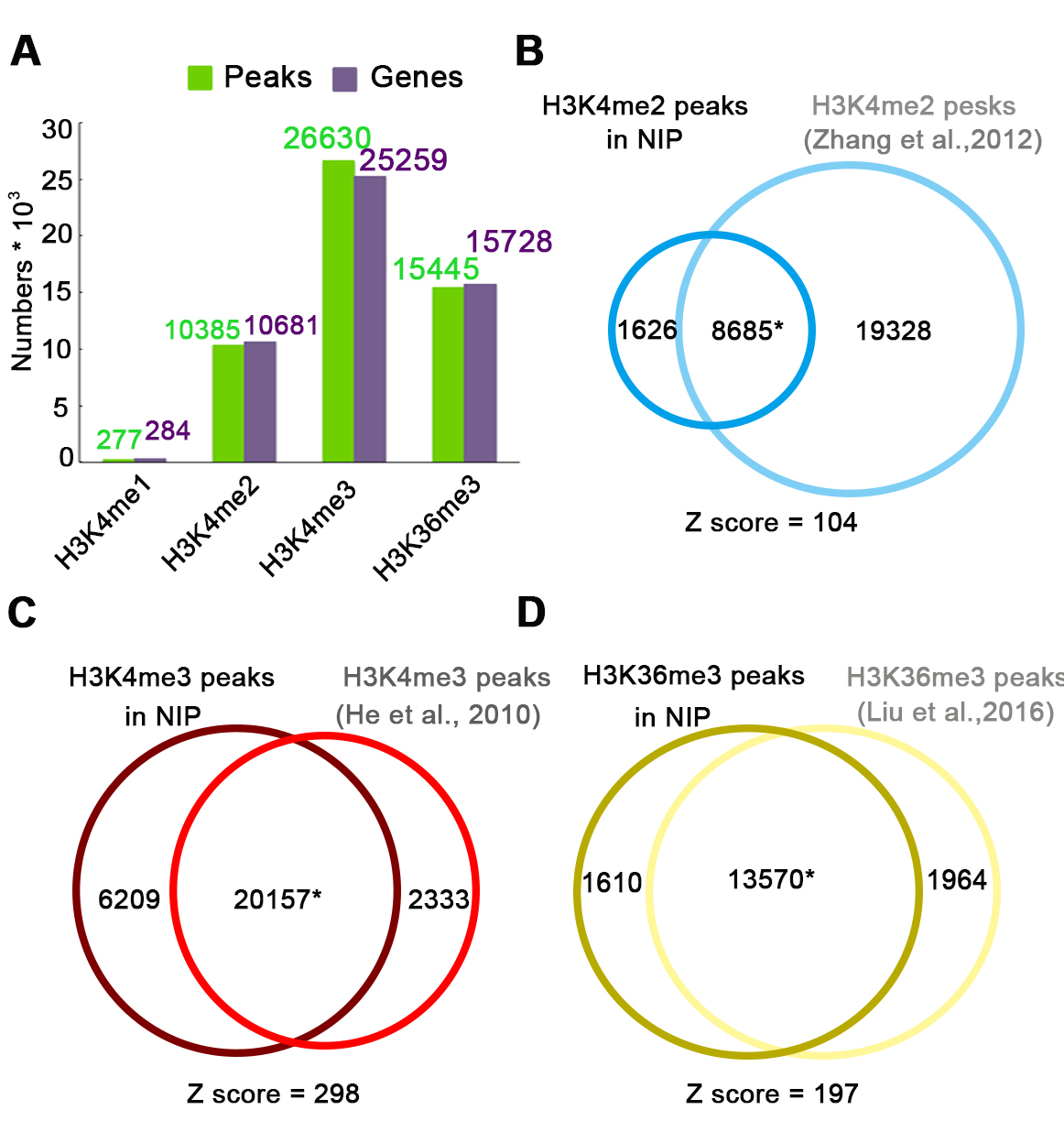
**

**Figure S2**. There was a considerable overlap between the ChIP-seq data in this study and previously published data.

A. Green and purple columns represent the peaks and genes, respectively, which were enriched with H3K4me1/me2/me3 and H3K36me3 in this study.

B–D. Venn diagrams presenting the significant overlap between the ChIP-seq data in this study and previously published data in the NIP background. A permutation test was used to evaluate the significance of the overlapping peaks (p = 0.0009).

**
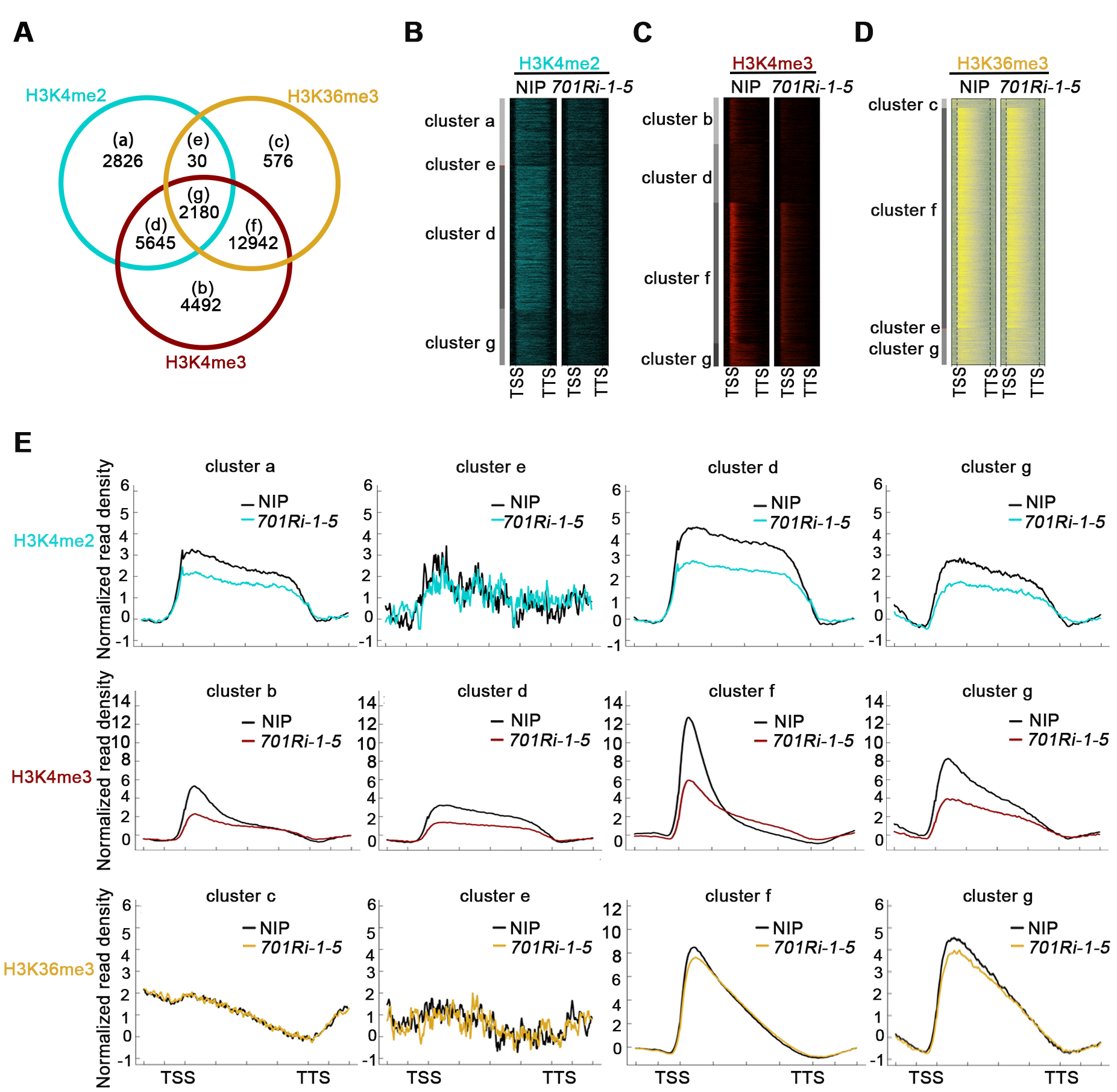
**

**Figure S3**. Decreased H3K36me3 levels in the *701Ri-1-5* mutant were not due to a lack of SDG701 activity.

A. Venn diagram presenting the number of genes significantly enriched with H3K4me2 (cyan), H3K4me3 (red), and H3K36me3 (yellow) in the wild-type NIP. Genes enriched with different marks were grouped into clusters a–g, and the number of genes in each cluster is indicated.

B–D. Heatmaps presenting H3K4me2 (B), H3K4me3 (C), and H3K36me3 (D) distributions at each gene locus of clusters a–g in NIP and the *701Ri-1-5* mutant.

E. The plot for each gene was generated from 1 kb upstream of the TSS to 1 kb downstream of the TTS. Average density plots presenting the H3K4me2 (top), H3K4me3 (middle), and H3K36me3 (bottom) levels for the genes in clusters a–g in NIP and the *701Ri-1-5* mutant.


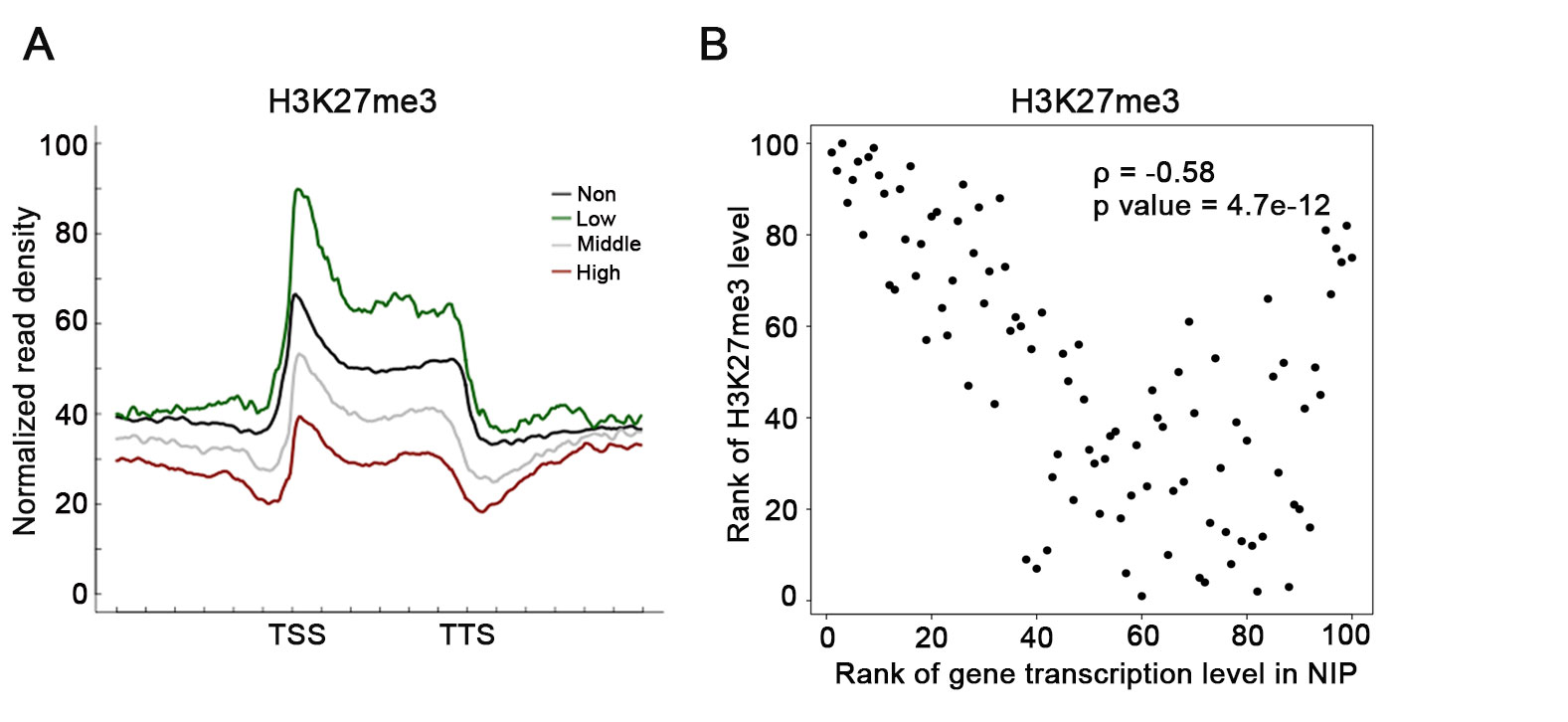


**Figure S4**. The H3K27me3 level was negatively correlated with the gene transcription level in rice.

A. For each gene, the plot was generated from 3 kb upstream of the TSS to 3 kb downstream of the TTS. Average density plots presenting H3K27me3 signals at differentially expressed genes in rice. Non (not expressed): 0 < FPKM < 1; Low (low expression level):1 ≤ FPKM < 2; Middle (moderate expression level): 2 ≤ FPKM < 10; and High (high expression level): FPKM ≥ 10.

B. Scatter plot presenting the relationship between the H3K27me3 level and the gene transcription level. The H3K27me3 level was calculated as follows: ChIP-seq normalized read density - input normalized read density for expressed genes (FPKM > 1). Spearman’s rank correlation coefficient indicates the correlation between the methylation level and the gene expression level.

**
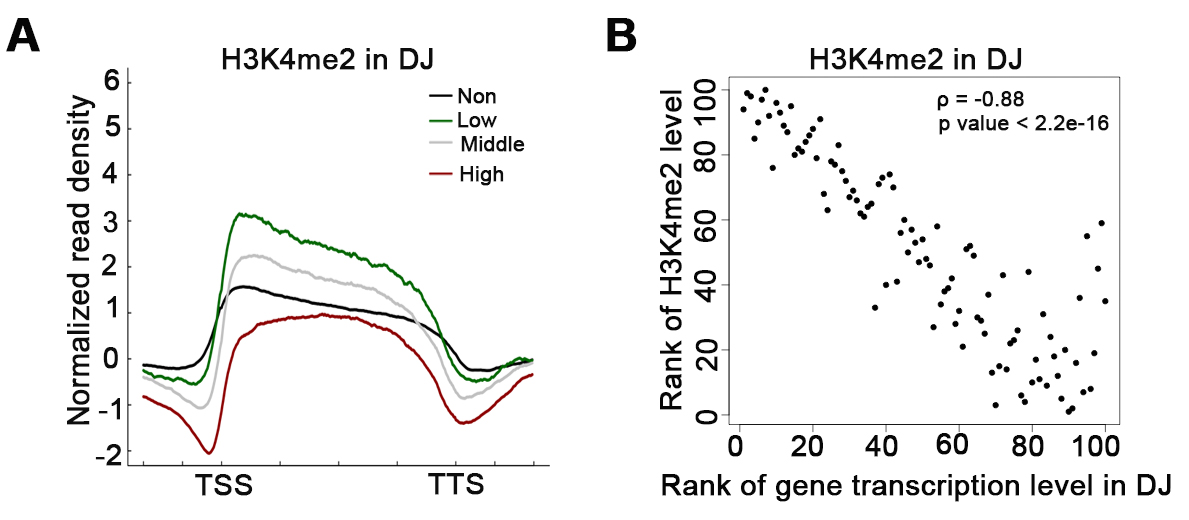
**

**Figure S5**. The H3K4me2 level was negatively correlated with the gene transcription level in the rice subspecies *Oryza sativa* ssp. *japonica* cv. Dongjin (DJ).

A. For each gene, the plot was generated from 1 kb upstream of the TSS to 1 kb downstream of the TTS. Average density plots presenting H3K4me2 signals at differentially expressed genes in DJ. Non (not expressed): 0 < FPKM < 1; Low (low expression level):1 ≤ FPKM < 2; Middle (moderate expression level): 2 ≤ FPKM < 10; and High (high expression level): FPKM ≥ 10.

B. Scatter plot presenting the relationship between the H3K4me2 level and the gene transcription level. The H3K4me2 level was calculated as follows: ChIP-seq normalized read density − input normalized read density for expressed genes (FPKM > 1). Spearman’s rank correlation coefficient indicates the correlation between the methylation level and the gene expression level.


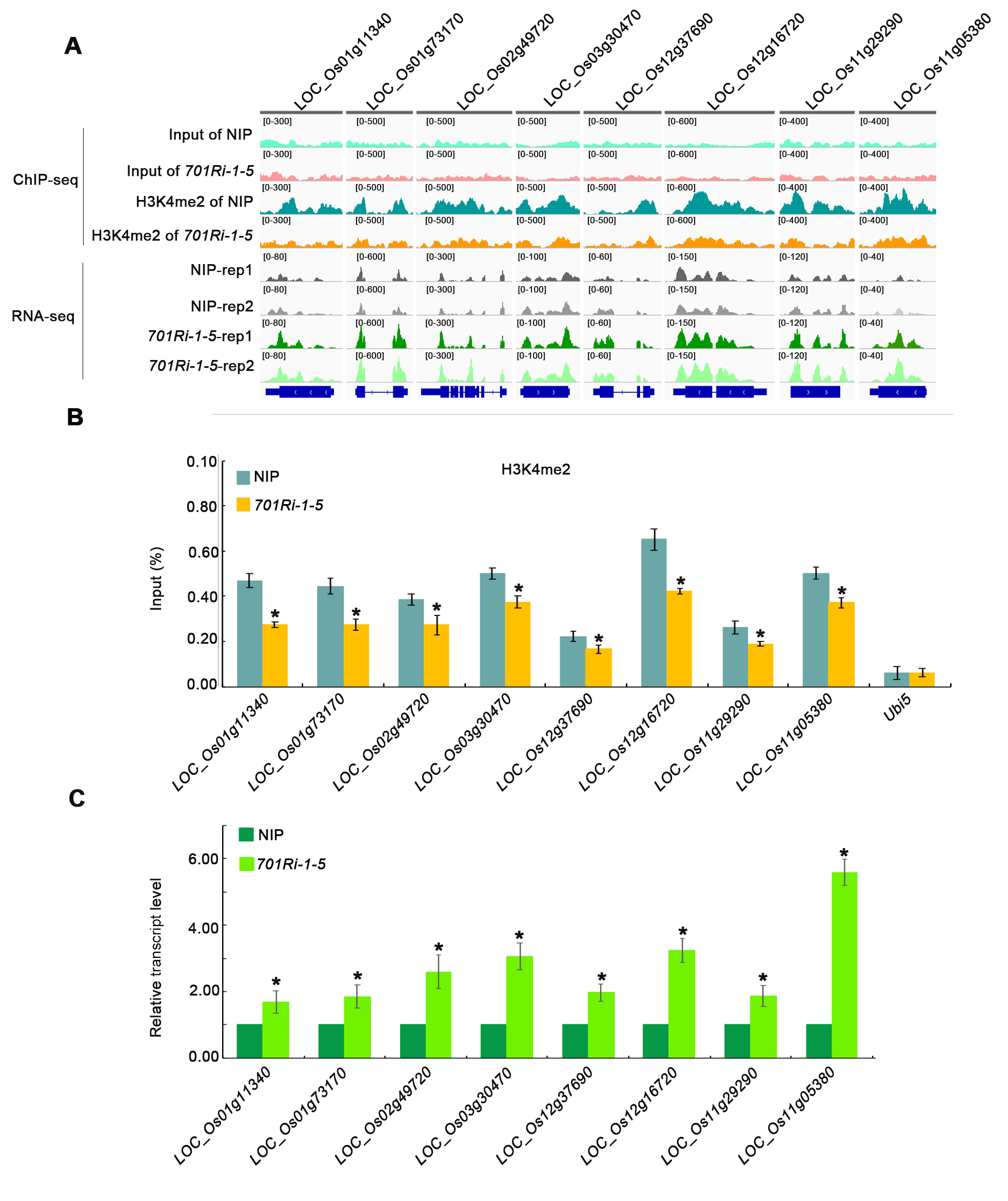


**Figure S6.** Validation of randomly selected genes with increased transcript and decreased H3K4me2 levels in the *701Ri-1-5* mutant.

A. ChIP-seq and RNA-seq tracks showing that the H3K4me2 levels and transcript levels of eight genes significantly decrease and increase in the *701Ri-1-5* mutant compared to in the wild-type NIP, respectively.

B. ChIP-qPCR results showing that the H3K4me2 levels of the selected genes significantly decrease in the *701Ri-1-5* mutant compared to in the wild-type NIP. Values are the mean ± SD of three individual biological replicates and *OsUbiquitin5* acts as an internal control. Asterisks indicate significant differences between *701Ri-1-5* and NIP (*P* < 0.01, Student’s t-test).

C. RT-qPCR results showing that the transcript levels of the selected genes were significantly up-regulated in the *701Ri-1-5* mutant compared to in the wild-type NIP. Values are the mean ± standard deviation (SD) from three independent biological replicates normalized to the internal control *OsUbiquitin5*. Asterisks indicate significant differences between *701Ri-1-5* and NIP (*P* < 0.01, Student’s t-test).

**
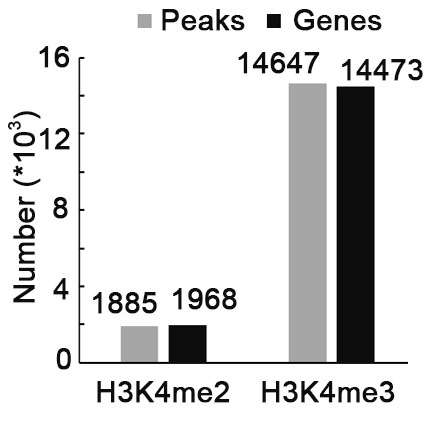
**

**Figure S7**. Genes with decreased levels of H3K4me2 and H3K4me3 in the *701Ri-1-5* mutant.

Gray and black columns represent the peaks and genes, respectively, with lower H3K4me2 and H3K4me3 levels in the *701Ri-1-5* mutant than in the wild-type NIP.

**
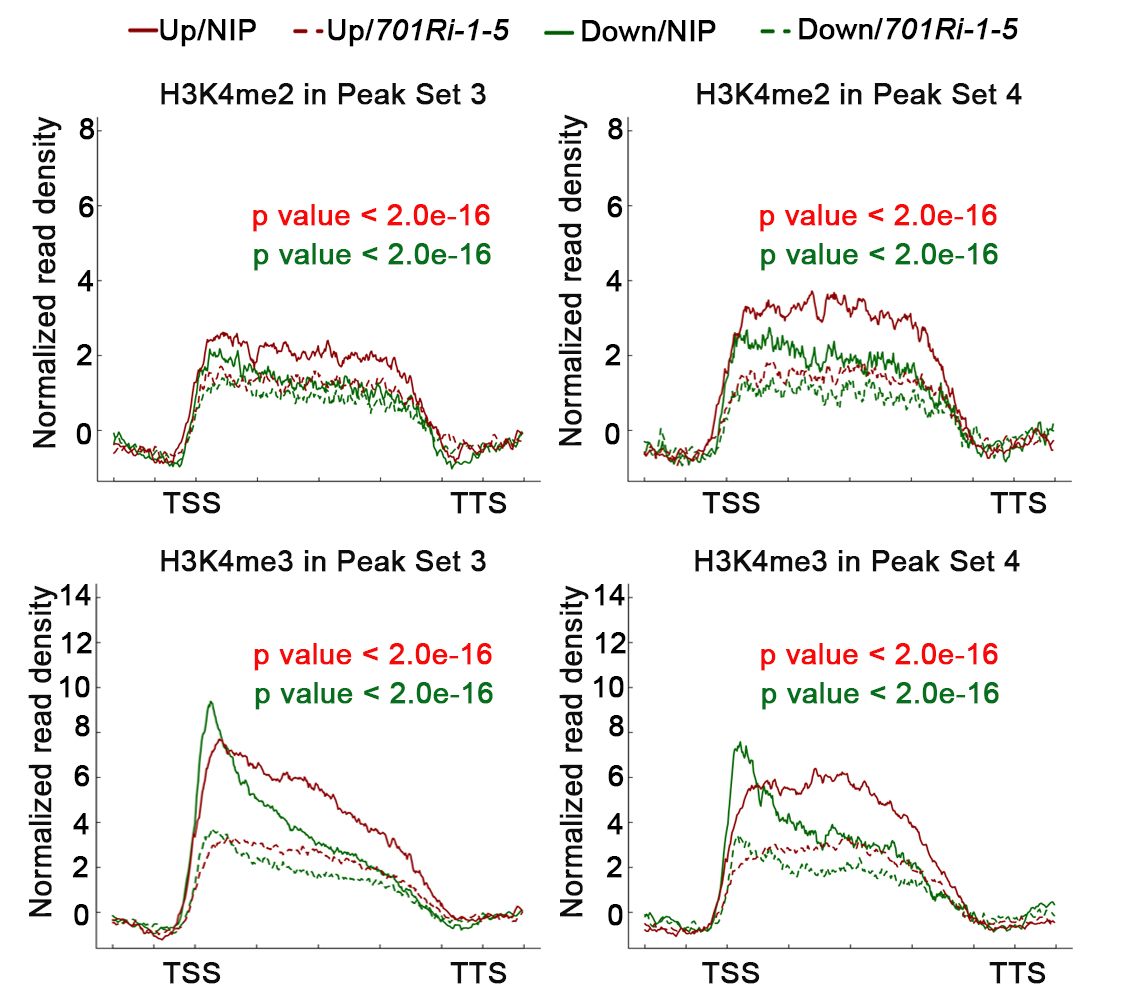
**

**Figure S8.** Average density plots of H3K4me2/me3 levels along the Peak Set 3 (left) and 4 (right) genes exhibiting up- (red) and down-regulated (green) expression, respectively.


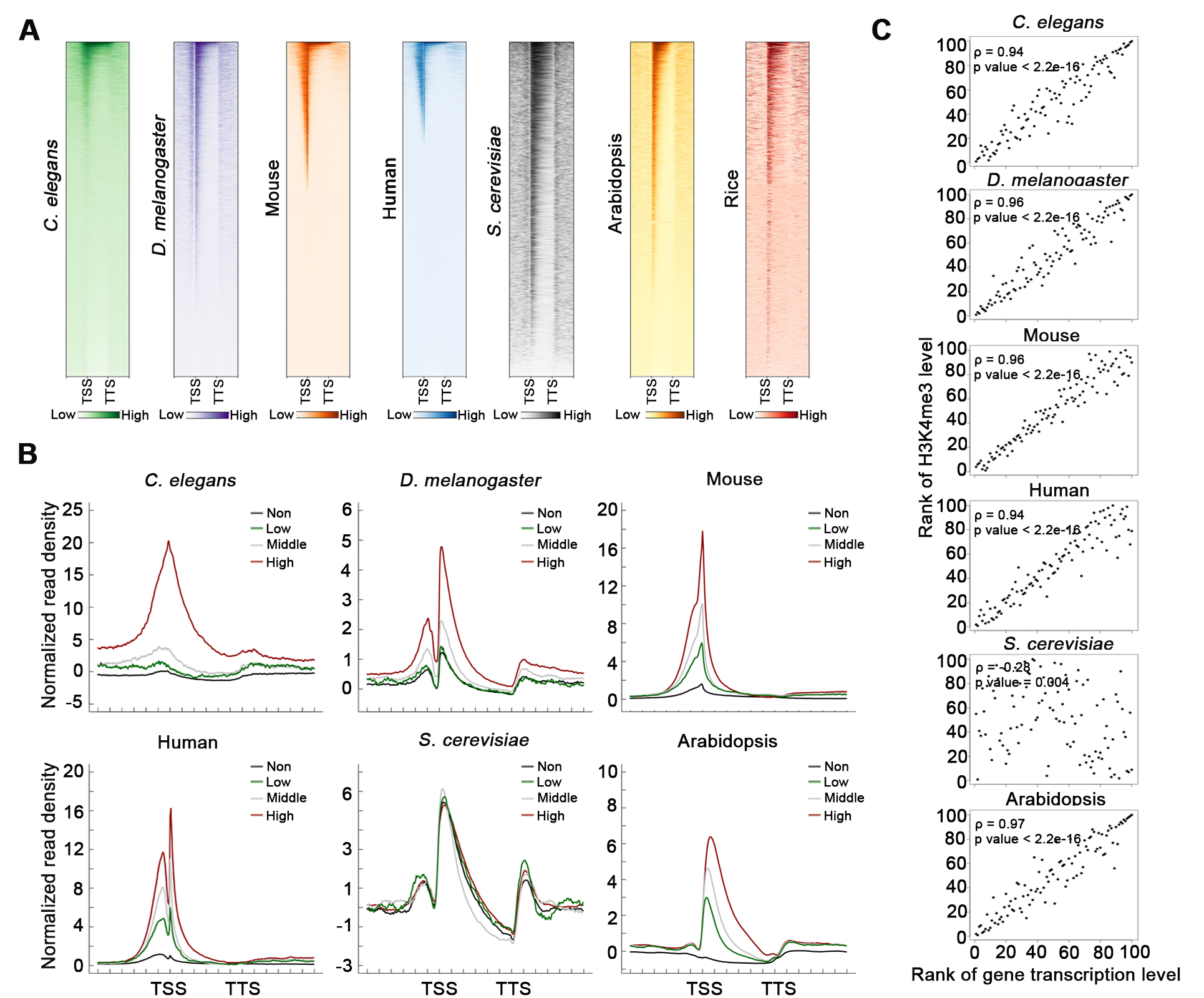


**Figure S9**. The H3K4me3 mark was enriched at the TSS and was positively correlated with gene expression in animals and plants.

A. Heatmaps presenting the H3K4me3 distributions in the genome of various species. For each gene, the plot was generated from 3 kb upstream of the TSS to 3 kb downstream of the TTS. From left to right: *Caenorhabditis elegans*, *Drosophila melanogaster* testis cells, mouse embryonic stem cells, human colon cancer cells (HCT116), *Saccharomyces cerevisiae*, Arabidopsis, and rice.

B. Average density plots presenting H3K4me3 signals at differentially expressed genes in various species. Non: 0 < FPKM < 1; Low: 1 ≤ FPKM < 2; Middle: 2 ≤ FPKM < 10; and High: FPKM ≥ 10.

C. Scatter plot presenting the relationship between the H3K4me3 level and the gene transcription level. The H3K4me3 level was calculated as follows: ChIP-seq normalized read density − input normalized read density for expressed genes (FPKM > 1). Spearman’s rank correlation coefficient indicates the correlation between the methylation level and the gene expression level.


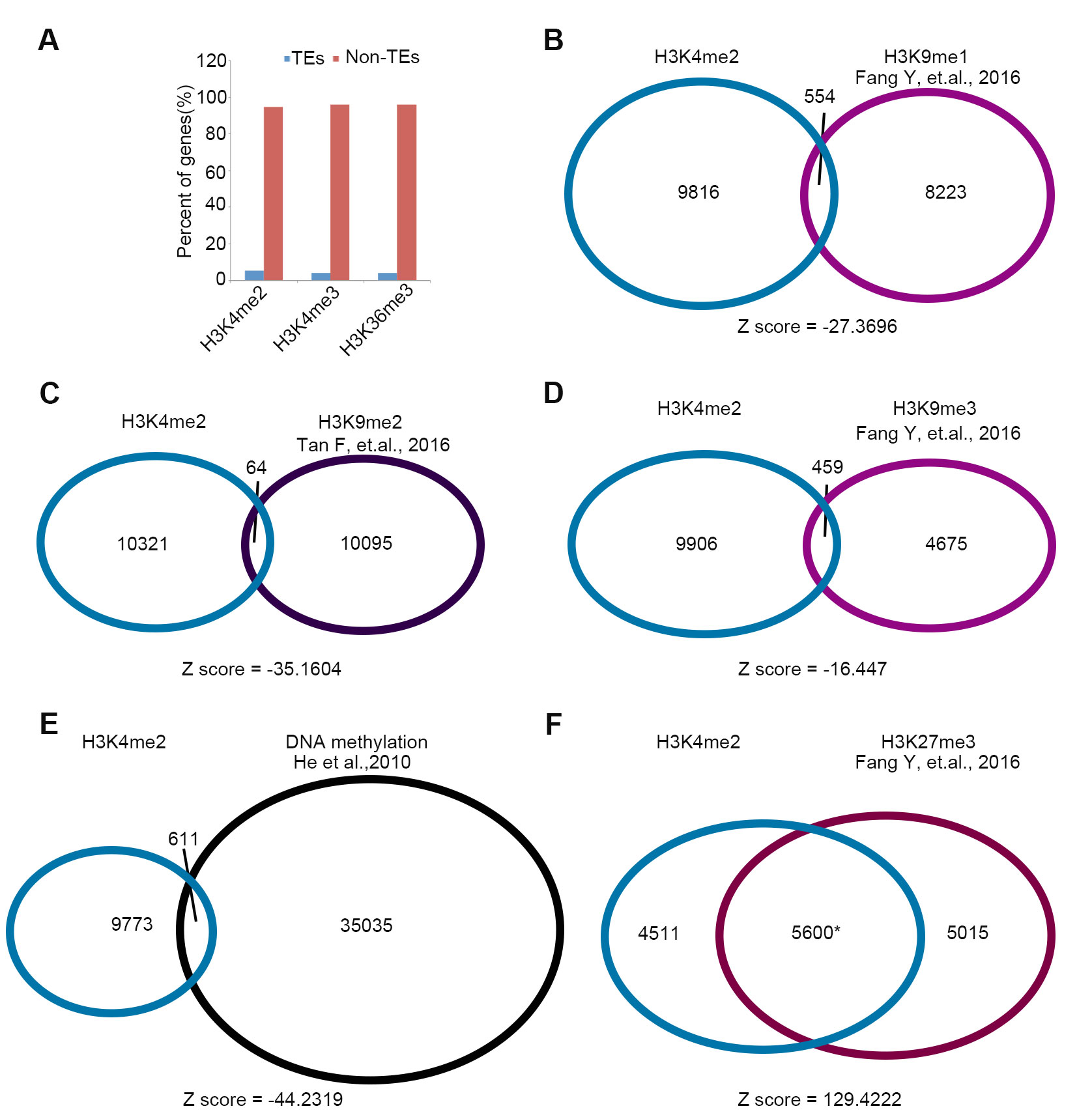


**Figure S10**. Peaks enriched with H3K4me2 significantly overlapped those enriched with H3K27me3 in rice.

A. The H3K4me2/me3 and H3K36me3 marks were mainly distributed within non-TE (transposable element) chromatin regions.

B–F. Overlap of the genes enriched with H3K4me2 and those enriched with H3K9me1 (B), H3K9me2 (C), H3K9me3 (D), DNA methylation (CG, CHH, and CHG) (E), and H3K27me3 (F) in rice. *Z score > 50 according to Fisher’s exact test, indicating H3K4me2-enriched regions significantly overlapped regions enriched with H3K27me3. A permutation test was used to evaluate the significance of the overlapping peaks (p = 0.0009).

**Table S1.** Summary of the RNA-seq mapping

| Sample | Raw reads | Mapped reads | % mapped reads |
| --- | --- | --- | --- |
| NIP-rep1 | 31576084 | 23597938 | 74.73 |
| NIP-rep2 | 42961148 | 32726819 | 76.18 |
| *701Ri-1-5*-rep1 | 34233346 | 27060342 | 79.05 |
| *701Ri-1-5*-rep2 | 38128820 | 30950689 | 81.17 |

**Table S4.** Summary of the ChIP-seq mapping

| Sample | Raw reads | Mapped reads | % mapped reads | Unique mapped reads | Non-redundant mapped reads |
| --- | --- | --- | --- | --- | --- |
| *701Ri-1-5*_H3K4me1 | 8027095 | 6292588 | 78.39 | 4384315 | 3905902 |
| *701Ri-1-5*_H3K4me2 | 12781414 | 12311470 | 96.32 | 8905836 | 8714359 |
| *701Ri-1-5*_H3K4me3 | 28865331 | 24162253 | 83.71 | 17699927 | 15210155 |
| *701Ri-1-5*_H3K36me3 | 21862619 | 20382643 | 93.23 | 15563008 | 14663537 |
| *701Ri-1-5*_input | 25505724 | 25122073 | 98.50 | 15942942 | 15588376 |
| NIP_H3K4me1 | 11983140 | 10250047 | 85.54 | 7091656 | 6328401 |
| NIP_H3K4me2 | 12793882 | 12249264 | 95.74 | 8863649 | 8659688 |
| NIP_H3K4me3 | 21424152 | 20606741 | 96.18 | 15797384 | 14497025 |
| NIP_H3K36me3 | 20962319 | 19382036 | 92.46 | 14855414 | 13861796 |
| NIP_input | 32443734 | 31814352 | 98.06 | 20274748 | 19661648 |

**Table S5.** Significant differences (SD) in the gene transcription level for each cluster in Figure 3B

| Group1 | Group2 | p value | SD | Method |
| --- | --- | --- | --- | --- |
| H3K4me2 | H3K4me2/H3K36me3 | 0.3388 | ns | Wilcoxon |
| H3K4me2 | H3K4me2/H3K4me3 | < 2e-16 | **** | Wilcoxon |
| H3K4me2 | H3K4me3 | < 2e-16 | **** | Wilcoxon |
| H3K4me2 | H3K36me3 | < 2e-16 | **** | Wilcoxon |
| H3K4me2 | H3K4me3/H3K36me3 | < 2e-16 | **** | Wilcoxon |
| H3K4me2 | H3K4me2/H3K4me3/H3K36me3 | < 2e-16 | **** | Wilcoxon |
| H3K4me2/H3K36me3 | H3K4me2/H3K4me3 | 0.4359 | ns | Wilcoxon |
| H3K4me2/H3K36me3 | H3K4me3 | 0.0001 | *** | Wilcoxon |
| H3K4me2/H3K36me3 | H3K36me3 | 5.60E-05 | **** | Wilcoxon |
| H3K4me2/H3K36me3 | H3K4me3/H3K36me3 | 4.20E-06 | **** | Wilcoxon |
| H3K4me2/H3K36me3 | H3K4me2/H3K4me3/H3K36me3 | 2.90E-11 | **** | Wilcoxon |
| H3K4me2/H3K4me3 | H3K4me3 | < 2e-16 | **** | Wilcoxon |
| H3K4me2/H3K4me3 | H3K36me3 | < 2e-16 | **** | Wilcoxon |
| H3K4me2/H3K4me3 | H3K4me3/H3K36me3 | < 2e-16 | **** | Wilcoxon |
| H3K4me2/H3K4me3 | H3K4me2/H3K4me3/H3K36me3 | < 2e-16 | **** | Wilcoxon |
| H3K4me3 | H3K36me3 | 0.6131 | ns | Wilcoxon |
| H3K4me3 | H3K4me3/H3K36me3 | 9.80E-06 | **** | Wilcoxon |
| H3K4me3 | H3K4me2/H3K4me3/H3K36me3 | < 2e-16 | **** | Wilcoxon |
| H3K36me3 | H3K4me3/H3K36me3 | 0.0016 | ** | Wilcoxon |
| H3K36me3 | H3K4me2/H3K4me3/H3K36me3 | < 2e-16 | **** | Wilcoxon |
| H3K4me3/H3K36me3 | H3K4me2/H3K4me3/H3K36me3 | < 2e-16 | **** | Wilcoxon |

**Table S8.** The gene-specific primers used in this study

| Gene | Primer | | Sequence | Assays |
| --- | --- | --- | --- | --- |
| LOC_Os01g11340 | g1-rt-F | GTCTCCGTCGTGGACTTCCG | | RT-PCR |
|  | g1-rt-R | CAGAGCAAGAGGACAAGGGA | |  |
|  | g1-IP-F | CCTCCTCCCTCTGCTGG | | ChIP-qPCR |
|  | g1-IP-R | TCGGCGGTGATGGGCT | |  |
| LOC_Os01g73170 | g2-F | GTGCCCGAAGAACGACAC | | RT-PCR & ChIP-qPCR |
|  | g2-R | CGAACTGGTGGAAGAACGCC | |  |
| LOC_Os02g49720 | g3-F | TTCGGCGGCTACAAGATGAG | | RT-PCR & ChIP-qPCR |
|  | g3-R | CGATGGGGATGGAGGAGAGA | |  |
| LOC_Os03g30470 | g4-F | CGGGCTGGAGTGCGG | | RT-PCR & ChIP-qPCR |
|  | g4-R | GCTGACGCCGAGGATGT | |  |
| LOC_Os12g37690 | g5-rt-F | ATGCCTCGCCTCGCC | | RT-PCR |
|  | g5-rt-R | CCCCACCATTCACCATCTCA | |  |
|  | g5-IP-F | GAAGGGTTGAAGAGGACGGG | | ChIP-qPCR |
|  | g5-IP-R | AGCGAGCGGAATAATGGGTG | |  |
| LOC_Os12g16720 | g6-F | GACGCTGGAGTGGGTGATG | | RT-PCR & ChIP-qPCR |
|  | g6-R | CGCCGAGGTGGGACT | |  |
| LOC_Os11g29290 | g7-F | TTGGAGCTGCCTCCTTGTGG | | RT-PCR & ChIP-qPCR |
|  | g7-R | AAGAAGATCCACAGGACGATGAA | |  |
| LOC_Os11g05380 | g8-F | GCCTACAAGATCATCGCCCA | | RT-PCR & ChIP-qPCR |
|  | g8-R | GATGTTGTCGAAGGCGAAGC | |  |
